# Optimizing filamentous fungi identification by MALDI-TOF MS: A comparative analysis of key factors

**DOI:** 10.1007/s10096-025-05316-0

**Published:** 2025-10-28

**Authors:** Özlem Dogan, Busra Betul Ozmen-Capin, John White, Chiara Serra, Sultan Ahmed, Silvia Botero-Kleiven, Volkan Özenci

**Affiliations:** 1 Division of Clinical Microbiology F2, Department of Laboratory Medicine, Karolinska University Hospital, Huddinge, 141 86 Stockholm SE Sweden; 2 Department of Medical Microbiology, Koc University, İstanbul, Turkey; 3 Department of Clinical Microbiology, Karolinska University Hospital, Huddinge, Sweden; 4 Department of Microbiology, Tumor and Cell Biology, Karolinska Institutet, Stockholm, Sweden; 5 Department of Medicine and Surgery, University of Perugia, Perugia, Italy

**Keywords:** MALDI-TOF MS, Filamentous fungi, Bruker biotyper, Sirius one, Microflex, MSI-2 database

## Abstract

**Purpose:**

The identification of filamentous fungi in clinical microbiology laboratories remains a challenging task. Although matrix-assisted laser desorption/ionization–time of flight mass spectrometry (MALDI-TOF MS) has revolutionized microbial diagnostics by enabling rapid and accurate species-level identification, its application to molds is still evolving. This study aims to evaluate the performance of two Bruker MALDI-TOF MS systems, Sirius One and Microflex 3.1, for the identification of filamentous fungi using different extraction protocols and database configurations.

**Method:**

A total of 68 filamentous fungal isolates, including clinically significant species, were analyzed. Fungal cultures were processed under standardized conditions using two protein extraction methods: a detailed in-tube extraction with ethanol, formic acid, and acetonitrile, and a direct on-plate extraction. Spectra were acquired using both Sirius One and Microflex 3.1 systems, and identifications were performed using manufacturer-provided databases and the MSI-2.0 database.

**Results:**

The Sirius One system outperformed Microflex 3.1, achieving a 92.6% correct identification rate with the MSI-2 database compared to 70.6% for Microflex (*p* < 0.01). When using manufacturer-provided databases, identification rates were lower: 51.5% for Sirius One and 41.2% for Microflex. Notably, the on-plate extraction method performed comparably to the in-tube method, achieving 94.1% accuracy with Sirius One and the MSI-2 database.

**Conclusion:**

The combination of the Sirius One system, MSI-2.0 database, and on-plate extraction method provides a highly effective and time-efficient workflow for the identification of filamentous fungi in routine clinical diagnostics, reaching 94.1% accuracy. This approach is recommended for implementation in clinical mycology laboratories, though further optimization of manufacturer-supplied databases remains necessary.

## Introduction

 The accurate and rapid identification of filamentous fungi remains a significant challenge in clinical mycology laboratories. Conventional identification methods require longer incubation times for accurate morphological identification. Since identification is made by macroscopic and microscopic examination, the experience of the personnel plays an important role. Even with the use of best laboratory practices, it is not possible to distinguish morphologically identical species belonging to the same species complex by microscopy alone. However, due to variable antifungal susceptibility profiles between different species within the same complex, it is critical to reach a definitive diagnosis as quickly as possible to apply the treatment correctly [1]. While morphological methods can be time-consuming and labor-intensive, modern diagnostic techniques have the potential to significantly improve this process. In this context, Matrix-Assisted Laser Desorption/Ionization Time-of-Flight Mass Spectrometry (MALDI-TOF MS) has revolutionized the routine identification of bacteria and yeasts and has shown promising results in the identification of filamentous fungi [2].

In the first studies in this field, the success rates in identifying filamentous fungi were found to be much lower than for bacteria and yeast [3]. In recent years, advancements in MALDI-TOF MS technology have enabled higher accuracy and faster identification of filamentous fungi [4]. Improved instruments and databases allow for more precise identification of clinically important fungal species. However, the need for a pre-extraction process to obtain better identification scores in filamentous fungi eliminates the advantage of definitive diagnosis within minutes offered by MALDI-TOF-MS in routine laboratories. To overcome this problem and accelerate accurate identification, Bruker has introduced the direct on-plate extraction method as an alternative to the time-consuming tube extraction method with its new Biotyper Sirius One system and MBT HT Filamentous Fungi IVD Module.

Within the newly established MBT HT Filamentous Fungi IVD Module, Bruker also enhanced the library content and algorithms for better identification accuracy in filamentous fungi [5]. In previous studies, older Bruker databases were found to be insufficient, and many researchers created their own in-house libraries or preferred to use online open access spectra analysis databases. For this purpose, one of the most frequently used online spectra analysis program is the Mass Spectrometry Identification (MSI) database [6]. Once, the MALDI-TOF analysis is completed in the instrument, the spectral data is extracted, then uploaded to the MSI system for library comparisons and identification. However, comparing the performance of different instruments, databases and extraction methods is critical for determining optimal diagnostic strategies.

This study aimed to compare the analytical performance of the Bruker MALDI Biotyper^®^ Sirius One system with the Bruker MALDI Biotyper Microflex 3.1 system in identifying clinically important filamentous fungi regarding protein extraction method and the use of different libraries.

## Materials and methods

### Sample collection

Selected filamentous fungi cultured from clinical samples and the quality control isolates (United Kingdom National External Quality Assessment Service, UK NEQAS) sent to the Mycology Section in the Department of Clinical Microbiology at the Karolinska University Hospital between 2020 and 2024 were included in the study (Table 1). When mold growth was observed in clinical samples, macroscopic and microscopic evaluation of the colonies and other conventional standard identification practices were performed [7]. Colonies were originally identified by using MALDI Biotyper Microflex 3.1 (Bruker Daltonics, Germany). If adequate identification could not be achieved with MALDI-TOF MS or if the results were inconsistent with conventional methods, isolates were then identified through NGS sequencing of the internal transcribed spacer (ITS) 1 and ITS2 regions and analyzed by using the UNITE database [8, 9]. The combination of all these identification methods defined the reference method used in this study. All fungal isolates were stored at −20 ℃ until the study.

**Table 1:** Species distribution of filamentous fungi included in the study library

Name	Species no	Name	Species no
Aspergillus spp	24	Order Mucorales	8
*Aspergillus fumigatus*	5	*Rhizopus oryzae/arrhizus*	3
*Aspergillus flavus*	3	*Rhizopus microsporus*	1
*Aspergillus terreus*	2	*Mucor circinelloides*	2
*Aspergillus nidulans*	2	*Lichtheimia ramosa*	1
*Aspergillus tubingensis*	3	*Syncephalastrum racemosum*	1
*Aspergillus niger*	1		
*Aspergillus lentulus*	1	***Talaromyces*** **spp**	**5**
*Aspergillus hiratsukae*	1	*Talaromyces columbinus*	1
*Aspergillus versicolor*	1	*Talaromyces flavovirens*	1
*Aspergillus calidoustus*	1	*Talaromyces atroroseus*	1
*Aspergillus citrinoterreus*	1	*Talaromyces wortmannii*	1
*Aspergillus termomutatus*	1	*Talaromyces ramulosus*	1
*Aspergillus sydowii*	1		
*Aspergillus montevidensis*	1	***Penicillium*** **spp**	**3**
		*Penicillium citrinum*	2
***Scedosporium*** **spp**.	**7**	*Penicillium chrysogenum*	1
*Scedosporium apiospermum*	3		
*Scedosporium aurantiacum*	2	***Purpureocillium lilacinum***	2
*Scedosporium boydii*	2		
		***Paecilomyces*** **spp**.	2
***Fusarium*** **spp.**	**7**	*Paecilomyces formosus*	1
*Fusarium verticillioides*	3	*Paecilomyces variotii*	1
*Fusarium proliferatum*	2		
*Fusarium dimerum*	2	***Acremonium*** **spp**.	2
*Fusarium petroliphilum*	1	*Acremonium egyptiacum*	1
***Lomentospora prolificans***	2	*Acremonium charticola*	1
***Scopulariopsis brevicaulis***	2	***Cladosporium pseudocladosporoides***	1
***Rasamsonia*** **spp.**	1	***Chaetomium bostrychodes***	1
***Trichoderma citrinoviridae***	1	**TOTAL**	**68**

Selected isolates for the study were sub-cultured from frozen stocks onto Sabouraud Dextrose Agar (SDA) plates, and all plates were incubated at 30 ℃. *Aspergillus* species and the members of the order Mucorales were incubated for three days, *Scedosporium* and *Fusarium* species for five days, and the remaining samples until visible sporulation or adequate biomass was observed. After the incubation period, the isolates underwent two different protein extraction methods. In our study, in-tube and on-plate extraction methods were used to compare their effectiveness in the identification of filamentous fungi.

### MALDI-TOF-MS preparation and extraction

#### In tube extraction method

**Fig. 1:**
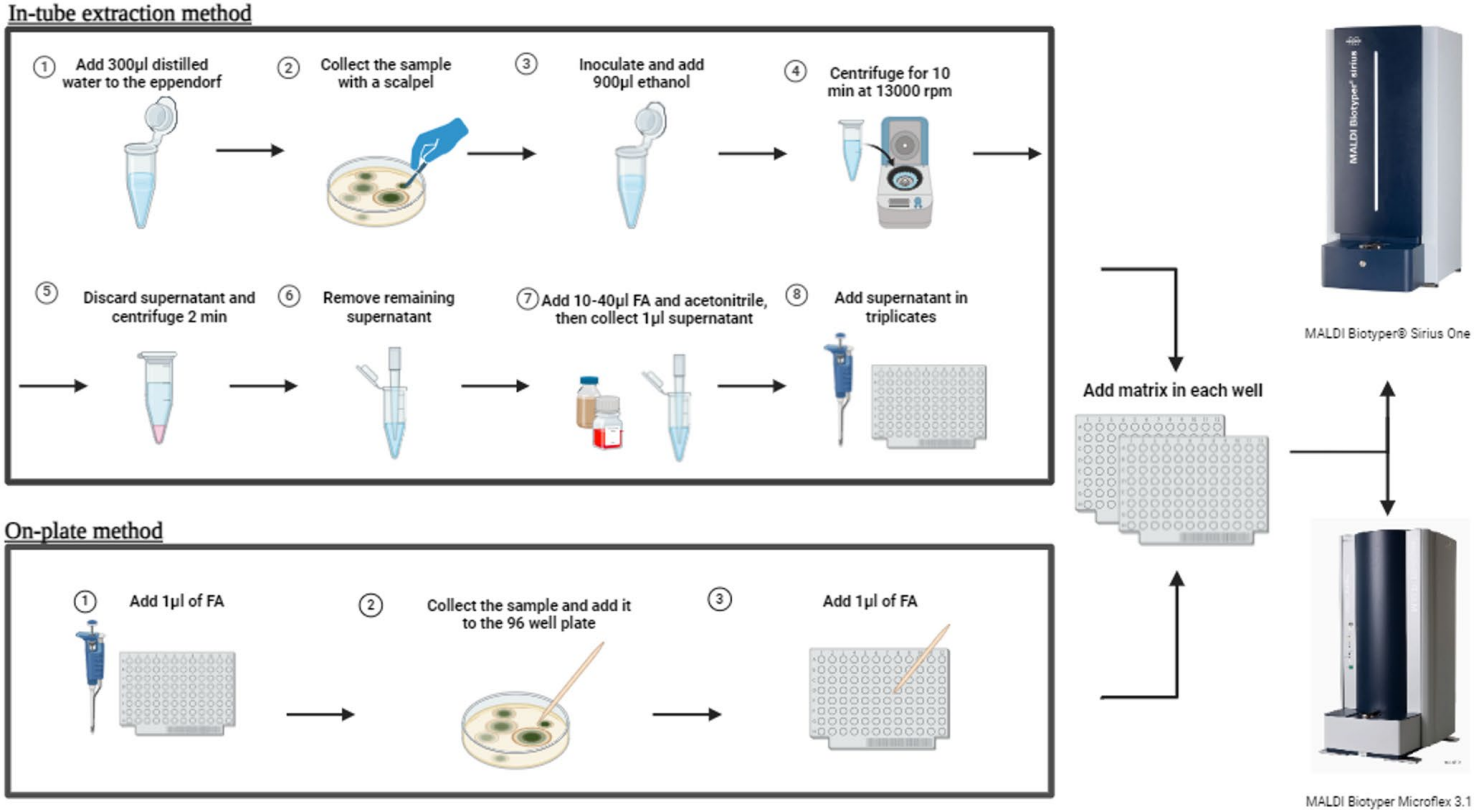
In-tube and on-plate extraction methods

For the identification of filamentous fungi using MALDI-TOF MS, the traditional tube extraction method was used according to the manufacturer’s recommendations. For this purpose, conidia and young mycelia from the edge regions of sufficiently mature colonies were collected using a scalpel and mixed with 300 µl of distilled water. Subsequently, 900 µl of 99% ethanol was added to the conidia suspension which was then centrifuged at 13,000 rpm for 10 min (Fig. 1). The supernatant was removed, and the remaining pellet was centrifuged again at 13,000 rpm for 2 min. The supernatant was removed again, and the remaining pellet was left to dry for 10 min. Then, depending on the density, 10–40 µl of 70% formic acid was added to the pellet. The suspension was left to stand for 5 min, after which an equal amount of acetonitrile was added. The mixture was centrifuged at 13,000 rpm for 2 min. 1 µl of the supernatant was added to the MALDI-TOF-MS plate. After complete drying, 1 µl of α-cyano-4-hydroxycinnamic acid (HCCA) matrix solution was added, and after drying at room temperature, the plate was placed into the MALDI-TOF instruments. The whole procedure for tube extraction took 45 min per isolate. After the extraction protocol isolates were spotted on plates in triplicates.

#### On-plate extraction method

In the first step of this method, 1 µl of 70% formic acid was added to the wells on the plate. Subsequently, conidia from the edge regions of sufficiently mature mold colonies were collected using a sterile wooden inoculation pick and mixed with the formic acid on the plate, and the mixture was left to dry at room temperature (Fig. 1). Once dried, another 1 µl of 70% formic acid was added to the wells and left to dry again. After the wells were completely dry, 1 µl of HCCA matrix solution was added, and the plate was allowed to dry again. Once the plate was completely dry, it was placed into the MALDI-TOF instruments. All experiments were performed in triplicates.

### MALDI-TOF MS analysis

In this study, the instruments of MALDI Biotyper^®^ Sirius One and the MALDI Biotyper Microflex 3.1 (both from Bruker Daltonics, Bremen, Germany) were used. Spectra were acquired for mold identification using the MBT HT Filamentous Fungi IVD Module on the MALDI Biotyper^®^ Sirius One system. Separately, the same samples were analyzed using the RUO database MBT Compass Library Revision K (2022) on the MALDI Biotyper^®^ Microflex 3.1 system, using the manufacturers standard settings. Since both systems utilize the same plates, the plates which were prepared with samples from two different extraction methods were first read with the MALDI Biotyper^®^ Sirius One System and then immediately passed through the MALDI Biotyper Microflex 3.1 system. The identified spectra were extracted from the two databases and transferred to the Mass Spectrometry Identification (MSI) Version-2.0 database for evaluation of the data with in-house database [6]. As the endpoint data, we had four different measurement points originated from the two versions of the manufacturer databases and the respective MSI-2 results for these spectra.

#### Interpretation criteria

The identification results were classified into one of the following categories: species level, complex/section level, genus level, no identification, and misidentification. Data from both MALDI-TOF MS instruments were analyzed as either correct or incorrect in comparison to results from the reference method. Correct identification was defined as matches at the species, genus, or complex/section level. Incorrect identification included results with no identification or misidentification [10]. Following the manufacturer’s recommendations, the MALDI Biotyper systems generate log scores of ≥ 2 for high confidence identifications, >1.7 for low confidence identifications, and ≤ 1.7 for no reliable confidence. In the case of the MSI-2 system, an identification was regarded as accurate only if the score reached at least 20. Misidentification was determined when the identification score exceeded the lowest confidence threshold, and the result did not match the reference identification.

#### Statistics

Statistical comparisons between the different extraction methods and the choice of reference library were performed in R version 4.4.1 using Rstudio 2024.04.2.764. The R CRAN packages psych, RVAideMemoire, and rcompanion were used [11–13]. Overall concordance between the different extraction and identification strategies were evaluated using Cochran’s Q-test and post-hoc pairwise comparisons were performed using McNemar’s test. P-values *≤* 0.05 were considered to be statistically significant. Cochran’s Q test and McNemar’s test are non-parametric statistical methods used to evaluate differences in paired binary outcomes, with McNemar’s test applied to two related groups and Cochran’s Q test extending the analysis to three or more related groups.

## Results

### Species distribution of study library

A total of 68 filamentous fungi representing 43 different species were included in the study. Among these, 47 were clinical isolates, while the remaining were quality control isolates. The species distribution of filamentous fungi included in the study library were presented in Table 1.

## Analytical performance of Bruker biotyper Sirius one and microflex 3.1 instruments

Overall, the percentage of correct identifications ranged from 30.9% to 94.1% between the different identification strategies. Choice of extraction method (on-plate vs. in-tube), instrument (Sirius vs. Microflex) and reference library (Bruker Filamentous Fungi vs. MSI-2) was found to significantly impact the level of successful identifications (p = < 2.2*10^−16^ Cochran’s Q-test).

The identification accuracy was higher in all three aspects with the new Biotyper Sirius One in comparison with Biotyper Microflex system. The correct identification rate with tube extraction method was higher in the new Biotyper Sirius One instrument (51.5%) compared with Biotyper Microflex (41.2%) when the data was analyzed with the manufacturers own libraries embedded in the systems, although it did not reach significance (*p* = 0.0923). Afterwards we extracted spectra from MBT database and analyzed via MSI-2, and the identification rates reached up to 92.6% with Biotyper Sirius One and 70.6% for Microflex (*p* < 0.001, Table 2).

**Table 2:** Diagnostic accuracy (%) of Bruker biotyper Sirius one and biotyper microflex with two different extraction methods

	On-plate Extraction			In-tube Extraction		
	Sirius One	Microflex	*P*-value	Sirius One	Microflex	*P*-value
Bruker Library	47% (32/68)	30.9% (21/68)	0.012	51.5% (35/68)	41.2% (28/68)	0.0923
MSI Library	94.1% (64/68)	58.8% (40/68)	< 0.001	92.6% (63/68)	70.6% (48/68)	< 0.001
	Sirius One			Microflex		
	On-plate Extraction	In-tube Extraction	*P*-value	On-plate Extraction	In-tube Extraction	*P*-value
Bruker Library	47% (32/68)	51.5% (35/68)	0.678	30.9% (21/68)	41.2% (28/68)	0.23
MSI Library	94.1% (64/68)	92.6% (63/68)	1	58.8% (40/68)	70.6% (48/68)	0.134

We observed the same identification rate differences also with on-plate extraction method. The correct identification rate for Biotyper Sirius One was 47% and 30.9% for Biotyper Microflex using the manufacturer libraries (*p* = 0.0127). The same spectra were also analyzed with the MSI-2 database, and the rates were found to be 94.1% with Biotyper Sirius One and 58.8% with Biotyper Microflex (*p* < 0.001). The MSI-2 database provided better results across all data points (Table 2).

The lowest identification rates were observed in isolates that underwent on-plate extraction, were analyzed using the Biotyper Microflex instrument, and had their spectra interpreted with the MBT Compass library. In contrast, the highest concordance was achieved in isolates also extracted on-plate but analyzed with the Biotyper Sirius One instrument and interpreted using the MSI-2 database (Table 2).

Interestingly, we observed no significant differences in the performance of on-plate or in-tube extraction for either MALDI instrument when using the same reference library system (Table 2). However, the in-tube extraction method yielded more consistent results (3 out of 3) compared to the on-plate method as 85.3% and 61.8% respectively (Table 3).

**Table 3:** The diagnostic accuracy of on plate and in-tube extraction method in triplicate experiments

	On-plate extraction accuracy (%)							
	Microflex (Own Library)		Sirius One (Own Library)		Microflex (MSI Library)		Sirius One (MSI Library)	
Mis ID	0		0		4.4		4.4	
No ID	69.1		52.9		36.8		1.5	
1/3	16.2		22		19.1		14.7	
2/3	10.3		16.2		23.5		17.6	
3/3	4.4		8.9		16.2		61.8	
	**100**,**0**		**100**,**0**		**100**,**0**		**100**,**0**	
		**In-tube extraction accuracy (%)**						
		**Microflex** **(Own Library)**		**Sirius One** **(Own Library)**		**Microflex** **(MSI Library)**		**Sirius One** **(MSI Library)**
Mis ID		0		1.5		0		5.9
No ID		58.8		47		27.9		1.5
1/3		8.8		4.4		10.3		1.5
2/3		10.3		5.9		11.8		5.9
3/3		22.1		41.2		50		85.2
		**100.0**		**100.0**		**100.0**		**100.0**

The MSI-2 identification system demonstrated a higher rate of misidentifications compared to Bruker’s proprietary system. Among these misidentifications, three involved *Aspergillus* species, notably the clinically significant *A. fumigatus*, *A. flavus*, and *A. nidulans*. Additionally, misidentifications were detected in species such as *Talaromyces ramulosus*, *Chaetomium bostrychodes*, *Scedosporium boydii* and *Acremonium charticola* (Table 4).

**Table 4.** The misidentified isolates and their identification scores in triplicate experiments

ID species	Sirius One/On-plate (Bruker Library)	Sirius One/On-plate (MSI Library)	Sirius One/In-tube (Bruker Library)	Sirius One/In-tube (MSI Library)	Microflex/On-plate (Bruker Library)	Microflex/On-plate (MSI Library)	Microflex/In-tube (Bruker Library)	Microflex/In-tube (Bruker Library)
*Aspergillus fumigatus*	0/3	1/3	3/3	3/3	0/3	*Sarocladium summerbellii*	3/3	3/3
*Aspergillus flavus*	1/3	*Aspergillus parvisclerotigenus/Exophiala spinifera*	0/3	*Microsporum audouinii*	2/3	0/3	0/3	0/3
*Aspergillus nidulans*	0/3	1/3	3/3	3/3	0/3	*Pseudopithomyces chartarum*	3/3	1/3
*Talaromyces ramulosus*	0/3	2/3	0/3	3/3	0/3	*Paecilomyces fulvus*	0/3	0/3
*Scedosporium boydii*	1/3	1/3	*Exophiala dermatidis*	*Exophiala dermatidis*	0/3	0/3	0/3	0/3
*Acremonium charticola*	0/3	*Keratinophyton indicum/Curvularia hominis/Keratinophyton durum*	0/3	*Thermoascus yaguchii*	0/3	0/3	0/3	3/3
*Chaetomium bostrychodes*	1/3	*Cryptendoxyla consimilis*	3/3	*Aspergillus protuberus/Chaetomium globosum*	0/3	0/3	1/3	0/3

## Discussion

MALDI-TOF MS is a highly useful tool for rapid and accurate identification of microorganisms in clinical settings. Studies have demonstrated its effectiveness in identifying common fungal species. However, challenges remain, such as the need for effective protein extraction methods and expanded databases [14, 15]. This study investigates the performance of the Sirius One and Microflex 3.1 Bruker MALDI-TOF MS systems, comparing in-tube and on-plate extraction methods for mold identification. It also evaluates the efficiency of the manufacturer’s library alongside the MSI-2 database, focusing on optimizing speed and accuracy for routine clinical applications.

This study included 68 distinct mold strains, featuring both clinical and quality control isolates and covering a wide spectrum of common filamentous fungi species identified in clinical samples. In our study, the Sirius One system significantly outperformed the Microflex 3.1, achieving 92.6% correct identification compared to 70.6% for Microflex (*p* < 0.01) for identification of filamentous fungi (Table 2). The MALDI Biotyper^®^ Sirius One System delivers quicker target exchange times compared to earlier models with a high-capacity vacuum pump. The advanced electronics also enable more rapid x/y-stage movements, significantly reducing time-to-result. To improve the detection time, Bruker also recommends on-plate extraction method for filamentous fungi identification combined with its MBT HT Filamentous Fungi Module for MALDI Biotyper Sirius One System.

Our study found that the on-plate extraction method performed equally well, and in some cases even outperformed, the conventional in-tube extraction method when tested with the Sirius One and Microflex Biotyper systems. There were no significant differences in analytical performance between the two approaches. (Table 2). However, in terms of speed, the on-plate method required only 10 min per sample compared to approximately 45 min for the in-tube method, highlighting a clear time advantage for routine clinical microbiology laboratory workflow. Choi et al. [10]. also compared the performance of the Bruker Biotyper, ASTA MicroIDSys, and VITEK MS systems in identifying 84 filamentous fungal isolates and they found that the in-tube extraction method provided the highest sensitivity, with the Bruker Biotyper achieving the best performance at a 71.43% correct identification rate. However, similar to our study, the researchers found no significant difference between the on-plate and in-tube extraction methods. We observed that, when using the in-tube extraction method, correct identification was more frequently achieved in all three replicates compared to the on-plate method. Although no formal reproducibility study was conducted, this observation suggests that performing tests in triplicate may increase the likelihood of obtaining at least one correct identification, particularly when using the on-plate extraction method. Therefore, incorporating triplicates may be beneficial for ensuring reliable results in routine laboratory practice.

A limitation of this study, is that the same plate which was first analyzed on the Sirius One was analyzed directly afterwards on the Microflex 3.1 system. Due to differences in system setups in number of shots fired at the plate when performing spectral analysis, the signal quality and strength in the Microflex 3.1 system was likely lowered. Therefore this could have led to a lower level of identification on the Microflex 3.1 system. In addition, while this study included several different species and genera from cryptic and non-cryptic species of fungi, our collection size was relatively small. Another limitation of this study is the potential geographical bias, as all isolates were collected from a single country, which may not fully represent the global diversity of filamentous fungi and caution should be taken when generalizing the findings to rare or region-specific species.

Another important aspect to consider when choosing a workflow, is the volume of single-use plastics involved. Single-use tips, tubes and scalpels are used to prevent cross contamination. However, in the modern hospital environment, action must be taken to reduce the use of unnecessary and non-biodegradable plastics. By adopting a primary workflow involving direct on-plate extraction before moving on to in-tube extraction, we can reduce our environmental footprint.

Among the two MALDI-TOF systems evaluated, the Sirius One demonstrated superior performance compared to the Microflex when using the MBT HT Filamentous Fungi IVD Module library, achieving higher identification rates. However, the improved library still could only identify approximately half of the isolates in this study. This highlights the need for enhanced solutions in filamentous fungi identification. To address these limitations, researchers have utilized in-house libraries or third-party databases, such as MSI-2, to enhance analytical performance. Normand et al. [6] evaluated the performance of the newly developed MSI-2 database in comparison to MSI-1 and the Bruker Filamentous Fungi Database for identifying molds. The study demonstrated that the MSI-2 database significantly outperformed both MSI-1 and Bruker in species-level identification, achieving an accuracy of 83.25%, compared to 63.19% for MSI-1 and 38.07% (1.7 threshold) for Bruker. The distinct advantage of using the MSI-2 database for the identification of filamentous fungi has also been demonstrated in various studies [16–19]. Notably, using the MSI-2 database enabled more accurate identifications in triplicate analyses, emphasizing its potential as a valuable tool in fungal diagnostics. Although the MSI system outperformed Bruker’s manufacturer library in species-level identification, it resulted in a higher number of misidentifications. Additionally, it should be recognized that the MSI-2 database is a non-IVDR certified reference library. Misidentifications missed by laboratory staff, for example, when a clinically relevant Rhizopus spp. is identified as e.g. Penicillium spp., this could result in delayed treatment, which could have devastating results on patient outcome. Therefore, results from the MSI-2 database should not be taken at face value but must be considered in conjunction with other microbiological data, such as microscopy, ideal temperature and macroscopic appearance. When all these findings are considered together, we propose a rapid, reliable, and environmentally friendly workflow for routine laboratories by utilizing the new MALDI-TOF Sirius One system combined with the on-plate extraction method.

Advancements in MALDI-TOF MS technology, such as the improved Sirius One system, have significantly enhanced diagnostic accuracy for filamentous fungi identification. The on-plate extraction method, with its efficiency and speed, demonstrates potential for incorporation into an algorithmic diagnostic workflow, especially in routine laboratory settings. However, the study highlights the importance of refining reference libraries to improve identification outcomes further. Incorporating third-party databases, like MSI-2, offers notable benefits and could provide a pathway to achieving even more reliable and comprehensive fungal diagnostics.

## Data Availability

The data that support the findings of this study are available from the corresponding author upon reasonable request.
